# Community-Based Follow-Up After NICU Discharge: A Practice-Focused Narrative Review of Nursing and Midwifery Care for Preterm Infants

**DOI:** 10.3390/pediatric18050116

**Published:** 2026-08-31

**Authors:** Vikentia Harizopoulou, Angeliki Bolou, Evdoxia Tsiakiri, Maria Bouroutzoglou, Victoria Vivilaki, Dimitra Metallinou

**Affiliations:** 1 Department of Midwifery, School of Health and Care Sciences, University of West Attica, 12243 Athens, Greece; abolou@uniwa.gr (A.B.); etsiakiri@uniwa.gr (E.T.); vvivilaki@uniwa.gr (V.V.); dmetallinou@uniwa.gr (D.M.); 2 Department of Midwifery, School of Health Sciences, International Hellenic University, 57400 Thessaloniki, Greece; mbmidwifery@gmail.com

**Keywords:** preterm infants, NICU discharge, community health nursing, midwifery, family-centred care, post-discharge follow-up, continuity of care, primary care, transition of care

## Abstract

Background/Objectives: Preterm birth remains a significant global public health challenge, associated with increased morbidity, rehospitalisation, and long-term vulnerability. The transition from the neonatal intensive care unit (NICU) to the home environment represents a critical phase. This practice-focused narrative review synthesises current evidence on community-based post-discharge follow-up for preterm infants, with particular emphasis on the complementary contribution of community health nursing and midwifery within multidisciplinary follow-up models, endocrine-metabolic surveillance and coordinated care for medically complex infants. Methods: A narrative synthesis of review articles, clinical guidelines, and empirical studies published between 2016 and 2026 was undertaken. Searches were performed in PubMed, Scopus, and CINAHL using terms related to preterm birth, NICU discharge, community follow-up, family-centred care, community health nursing, midwifery, and metabolic disorders. Results: Community-based, family-centred follow-up programmes are associated with improved continuity of care, increased parental confidence, enhanced breastfeeding outcomes, and reduced healthcare utilisation. Community health nurses, midwives and health visitors contribute complementary expertise within multidisciplinary follow-up by coordinating continuity of care, providing clinical surveillance of growth and feeding, supporting caregiver education, facilitating early identification of complications, and ensuring timely referral. However, endocrine and metabolic vulnerabilities remain underrepresented in many follow-up models, while infants with complex healthcare needs require individualised, risk-stratified surveillance. Conclusions: Community-based, interdisciplinary, and family-centred follow-up after NICU discharge is essential to enhance the quality and safety of care for preterm infants. Integrated, risk-stratified models of follow-up that combine preventive surveillance, family education, multidisciplinary coordination, and continuity of care may facilitate earlier recognition of complications and improve long-term outcomes.

## 1. Introduction

Preterm birth, defined as delivery before 37 completed weeks of gestation, affects an estimated 13.4 million infants worldwide annually and remains a leading cause of neonatal morbidity and mortality [1]. While recent advances in neonatal intensive care have resulted in survival rates ranging from 90% to 95% for many preterm infants [2,3], survival alone is insufficient as a marker of success [4]. Higher survival rates are accompanied by persistent acute complications and long-term sequelae extending well beyond the neonatal period, necessitating a broader focus on quality of life and ongoing care [4,5]. Accordingly, improving survival must be accompanied by strategies that reduce morbidity, support optimal development, and lessen the long-term burden on families.

Discharge from the neonatal intensive care unit (NICU) marks a distinct period of vulnerability and stress, characterised by complex healthcare needs, increased parental anxiety, and a heightened risk of rehospitalisation [6,7,8,9]. This transition is a dynamic process during which parents assume primary responsibility for their infant’s care while managing emotional distress, uncertainty, and evolving healthcare needs [3,6,8]. Despite structured discharge planning, many families continue to report feeling insufficiently prepared to care for their infant at home [6,10,11]. Furthermore, socioeconomic factors stratify risk; infants covered by public insurance or from families with limited English proficiency experience higher rates of rehospitalisation and face greater barriers to follow-up care [4,12].

To address these challenges, a life-course health development approach is recommended, emphasising integrated, family-centred follow-up care that addresses longitudinal medical and developmental needs [4]. Comprehensive discharge planning and early home-based support are essential to facilitate a safe transition and reduce avoidable readmissions [12,13,14]. The nomenclature and professional scope of those providing community-based follow-up vary internationally. Depending on the national context, health visitors, community health nurses, or community midwives may undertake this pivotal role [3,15]. Throughout this review, these terms are used inclusively to acknowledge the diversity of international models of post-discharge care while referring to the frontline professionals delivering community-based follow-up.

Effective community-based follow-up is inherently multidisciplinary. Depending on the organisation of national healthcare systems, services may involve community health nurses, specialised paediatric nurses, community midwives, health visitors, paediatricians, physiotherapists, occupational therapists, speech and language therapists, psychologists, dietitians, social workers, and other healthcare and social care professionals [3,5,14]. Rather than representing overlapping professional roles, these disciplines provide complementary expertise within multidisciplinary follow-up models across the continuum of post-discharge care, supporting infant development, parental wellbeing, and continuity of care. Although professional responsibilities differ internationally, community health nurses, midwives, and health visitors are consistently recognised as key providers of longitudinal continuity of care, coordinating communication across services, supporting families at home, reinforcing parental confidence, and facilitating early recognition of clinical concerns that require referral or specialist input [3,11,15]. Working collaboratively with paediatricians and other professionals, they translate hospital recommendations into everyday practice, strengthen caregiver competence, facilitate coordinated family-centred follow-up, and reduce fragmentation across the care pathway [3,5,11,13,14].

Additionally, growing evidence underscores the importance of structured surveillance for endocrine and metabolic disturbances associated with prematurity [9,16,17]. Despite their implications for growth, bone health, and long-term cardiometabolic outcomes, these disorders remain inconsistently addressed within routine community follow-up, although opportunities exist for early identification through structured surveillance and nutritional support [4,17,18].

While numerous reviews have explored NICU follow-up and early intervention programmes, they often focus on neurodevelopmental outcomes from a medical or tertiary-care perspective [4]. Existing literature gives comparatively little attention to the complementary contributions of community health nurses, midwives, and health visitors within multidisciplinary follow-up models [3,11,15]. Accordingly, this narrative review provides a practice-focused conceptual synthesis of how community nursing and midwifery professionals contribute complementary expertise across the continuum of post-discharge care through family-centred support, multidisciplinary care coordination, early recognition of complications, and risk-stratified care for preterm infants with routine and complex healthcare needs.

## 2. Materials and Methods

### 2.1. Review Design

A narrative review methodology was selected because the available literature is heterogeneous, including clinical guidelines, review articles, qualitative research, observational studies, and intervention studies. This review was conducted in accordance with the recommendations for narrative literature reviews described by Ferrari (2015) [19] and reported following the SANRA (Scale for the Assessment of Narrative Review Articles) guidance proposed by Baethge et al. (2019) [20]. Unlike a scoping review, which primarily maps the breadth of available evidence, a narrative review was considered more appropriate because the objective was to develop a practice-focused conceptual synthesis of diverse evidence relevant to community-based follow-up after NICU discharge.

### 2.2. Search Strategy

Electronic searches were conducted in February 2026 in PubMed, Scopus, and CINAHL using Medical Subject Headings (MeSH), where applicable, together with keywords related to preterm infants, NICU discharge, community follow-up, transition of care, continuity of care, family-centred care, community nursing, midwifery, primary care and clinically relevant aspects of post-discharge follow-up. Two reviewers (V.H. and A.B.) performed the literature searches independently. Priority was assigned to articles published between 2015 and 2026, although earlier landmark studies were included when foundational to current practice.

### 2.3. Eligibility Criteria

Eligible publications were peer-reviewed articles published in English that addressed community-based follow-up of preterm infants after NICU discharge. Reviews, position statements, and clinical guidelines from high-income healthcare systems were prioritised because they reflected models relevant to European and comparable contexts. Studies focusing exclusively on inpatient NICU care without a post-discharge component, term infants or adult populations, conference abstracts, editorials, dissertations, grey literature, and non-English publications were excluded.

### 2.4. Study Selection

**Figure 1:**
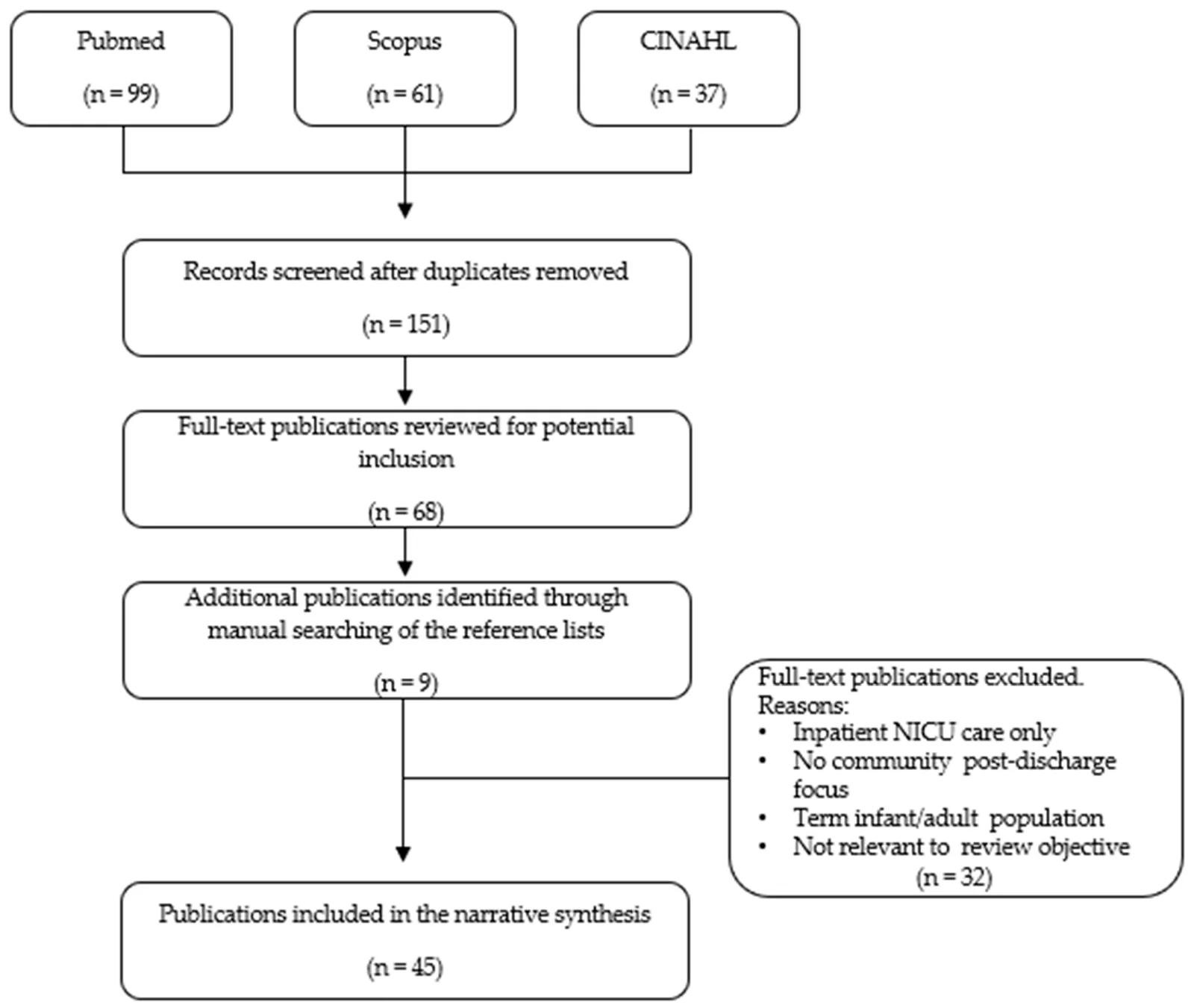
PRISMA-style flow diagram of the literature identification and selection process.

Study selection was undertaken in two stages. Initially, two reviewers (V.H. and A.B.) independently screened the titles and abstracts of all retrieved records against the predefined eligibility criteria. Full-text articles of potentially eligible studies were subsequently retrieved and independently assessed for inclusion. Disagreements were resolved through discussion and, when necessary, consultation with a third reviewer. A PRISMA-style flow diagram (Figure 1) summarises the literature identification, screening, eligibility assessment, and inclusion process.

### 2.5. Evidence Appraisal and Data Synthesis

Original observational and interventional studies were considered alongside reviews, clinical guidelines, and position statements to provide both the broader evidence base and contemporary examples of clinical practice. Consistent with narrative review principles, no formal risk-of-bias assessment was undertaken. Instead, the included literature was critically evaluated for methodological robustness, relevance to the review objectives, level of evidence and implications for community nursing and midwifery practice, with greater emphasis placed on high-level evidence and internationally recognised clinical guidance. Qualitative studies were included to provide contextual insight into parental experiences and implementation challenges.

The findings were synthesised using an iterative narrative approach. The included literature was read to identify recurring concepts, transitional challenges, and practice implications. Concepts were compared across studies, grouped by conceptual similarity, and refined through consensus among the authors into the overarching themes presented in the Results.

## 3. Results

The research identified 197 records, of which 45 publications met the eligibility criteria and were included in this narrative review (Figure 1). The included literature comprised clinical guidelines, systematic and narrative reviews, position statements, randomised controlled trials, observational studies, and qualitative research published between 2015 and 2026. Using an iterative narrative synthesis, the evidence was conceptually organised into five complementary themes reflecting the principal domains of multidisciplinary community-based follow-up after NICU discharge: (1) morbidity and rehospitalisation; (2) transition planning and psychosocial support; (3) interdisciplinary community care; (4) endocrine and metabolic surveillance; and (5) follow-up of infants with complex neonatal conditions. Collectively, these themes illustrate how community nursing and midwifery professionals contribute, alongside the wider multidisciplinary team, to continuity of care, family-centred support, coordinated follow-up, and improved long-term outcomes for preterm infants and their families.

### 3.1. Morbidity and Rehospitalisation

While the elevated risk of rehospitalisation for preterm infants is well documented [7,13], its implications for community practice are substantial. With readmission rates reaching up to 30% for high-risk infants within the first 90 days post-discharge [12], the immediate post-discharge period demands a shift from passive observation to proactive, community-based surveillance delivered within multidisciplinary follow-up models [3,4]. Because respiratory illnesses (particularly RSV and bronchopulmonary dysplasia), infections, and feeding difficulties drive most readmissions [7,11,12], community health professionals, particularly community nurses, midwives and health visitors, should prioritise targeted preventive interventions. Effective community follow-up extends beyond routine growth monitoring to include parental education on infection control, feeding assessment, and early recognition of clinical deterioration, thereby helping to prevent avoidable emergencies [11,14,21].

Furthermore, the chronic sequelae of prematurity, such as neurodevelopmental impairments and complex physiological conditions [1,4,22], transform the role of community nursing and midwifery professionals into one of essential care coordination. Consequently, many survivors are discharged with complex medical needs and technological support [22], translating into a sustained financial and psychological burden for families [7,23]. Therefore, effective community follow-up requires a longitudinal approach that addresses evolving clinical risks, coordinates care across services, and mitigates the cumulative strain on caregivers navigating complex care at home.

### 3.2. Family-Centred Transition Planning and Phychosocial Support

Post-discharge safety depends on structured transition planning that begins well before discharge and involves close collaboration between neonatal and community healthcare professionals [11,14]. By incorporating structured transition tools, such as “NICU Roadmaps” or the “Train-to-Home” intervention, into discharge planning, community health professionals can support safer transitions, as these interventions have been associated with reduced emergency department attendance [2,3,14]. Furthermore, because gaps in information sharing can lead to missed opportunities for early support [3,14], the available evidence supports establishing robust community "reception protocols" to ensure continuity of care upon the infant’s return home [14]. In practice, implementation of these reception protocols should extend beyond routine postnatal assessments to include comprehensive clinical and phychosocial evaluation [11,22,24]. An initial comprehensive home assessment enables community professionals to identify early clinical “red flags” (e.g., apnoea, cyanosis, poor feeding, lethargy), as well as conduct a rigorous, systematic medication review and a detailed feeding evaluation [3,12,22,24]. Additionally, practitioners can coach parents on infection prevention, providing explicit guidance on hand hygiene and visitor limitation to mitigate the high risk of community-acquired infections, including RSV [3,12,14].

Because this transition is frequently complicated by profound parental stress, unpreparedness, and even post-traumatic stress disorder [10,11,25], routine assessment of parental mental health represents an important component of comprehensive post-discharge follow-up [3,11,14]. Treating caregiver well-being as a safety parameter is essential, as an overwhelmed parent cannot effectively manage complex infant needs [11]. To mitigate these psychosocial challenges, family-centred support should be operationalised through structured, strengths-based interventions [4,10,26]. This requires professionals to adopt strengths-based interventions, such as those inspired by the Stockholm Preterm Interaction-Based Intervention (SPIBI), to actively coach parents in interpreting subtle infant cues, thereby building parental self-efficacy and reducing emotional distress [10,27]. Furthermore, evidence dictates a shift from fragmented, reactive care to proactive, structured follow-up [4,14,28]. Establishing relational continuity and providing sustained, proactive support via structured telephone calls or home visits directly enhance clinical metrics, such as breastfeeding rates and parental confidence [3,10,21,29]. By fostering active parental involvement, community nurses, midwives, and health visitors operationalise the Life Course Health Development (LCHD) framework into daily practice, promoting caregiver autonomy [3,4,30].

### 3.3. Multidisciplinary Community Care

Delivering coordinated care is frequently hindered by system-level challenges, including heterogeneity in follow-up services and premature cessation of specialist surveillance [4,31]. Because community-based follow-up of preterm infants is inherently multidisciplinary, community health nurses, midwives, and health visitors are well positioned to coordinate between families and the wider multidisciplinary team, facilitating communication, continuity of care, and timely referral across healthcare settings [3,4]. Rather than managing developmental concerns in isolation, these professionals can identify emerging problems early and initiate timely referrals to appropriate specialists, such as physiotherapists for infants at risk of cerebral palsy or speech-language pathologists for persistent feeding difficulties, thereby enabling earlier intervention and improving developmental outcomes [32].

Because responsibility for the infant’s health shifts largely to primary care upon discharge, community professionals often become the most consistent point of reference for families navigating uncertainty [3,4,33]. To operationalise the “medical home” concept, community practitioners are well positioned to coordinate care [4]. Within this collaborative framework, community midwives primarily facilitate the immediate post-discharge transition by safeguarding neonatal physiological stability and supporting maternal postpartum recovery and well-being [3,34,35], whereas community health nurses and health visitors provide longitudinal follow-up [3,11], including growth monitoring using corrected age [17,34], management of complex feeding regimens (including home enteral tube feeding) [15], developmental surveillance, and immunisation according to chronological age [3,14,32]. However, translating this role into practice is often hindered by a well-documented disconnect between tertiary NICU care and the community [3,11,15]. Effective communication, shared care plans, and clear referral pathways between neonatal and community services are fundamental to coordinated care [14,32]. When system-level integration falls short, community healthcare professionals become the primary coordinators of care for navigating complex services. Poor coordination contributes to fragmented care, parental distress, and avoidable use of acute healthcare services [28,31].

Early structured home-visiting programmes represent an effective strategy for reducing fragmentation and supporting families after discharge [8,21,33]. By addressing parental uncertainty directly in the home setting, specialised home-visiting teams led by community nurses and midwives can reduce parental stress, increase breastfeeding rates, and prevent avoidable emergency department visits [3,12,21]. Delivering effective home care requires professionals to actively cultivate empathetic, continuous relationships, as technical competence alone is insufficient [3]. Parents consistently value professionals who provide specific, preterm-tailored guidance, whereas perceived gaps in specialised knowledge may undermine trust [15]. This proactive approach is globally adaptable: trained nurses and midwives providing home care in resource-limited settings have reduced neonatal hospitalisations [24]. Ultimately, effective multidisciplinary community follow-up depends on community nurses, midwives, and health visitors integrating clinical surveillance, care coordination, and family-centred support to ensure continuity of care after NICU discharge [11,21,35].

### 3.4. Endocrine and Metabolic Surveillance

Endocrine and metabolic surveillance represents an important, yet comparatively underrepresented, component of community follow-up for preterm infants. Across the literature, endocrine and metabolic sequelae receive considerably less attention than respiratory and neurodevelopmental outcomes, despite their important long-term clinical implications [17,34,36,37]. This imbalance highlights the need to broaden multidisciplinary community follow-up beyond traditional priorities to include distinct endocrine and metabolic surveillance [17,34].

One critical yet frequently overlooked area is thyroid function. Preterm infants are particularly susceptible to transient hypothyroxinemia of prematurity and delayed thyroid-stimulating hormone (TSH) elevation [36,37]. Because initial neonatal screening in the NICU may not capture delayed-onset hypothyroidism, community nurses, midwives and health visitors should remain vigilant [16]. In practice, this means actively assessing for subtle clinical signs in the home environment, such as unexplained lethargy, poor feeding, prolonged jaundice, or hypotonia, and ensuring that scheduled repeat thyroid function tests are completed, rather than assuming the initial newborn screening was definitive [16,36,37].

Given the increased risk of impaired calcium and phosphorus accretion in preterm infants, community practitioners play an important role in assessing adherence to prescribed supplementation (e.g., human milk fortifiers, calcium/phosphorus) and collaborating with paediatricians to support appropriate biochemical monitoring, including alkaline phosphatase and serum phosphorus [17,34]. Furthermore, because osteopenia of prematurity severely compromises bone density and increases the risk of atraumatic fractures [18], community professionals can educate parents on safe infant handling, lifting, and bathing, translating these risks into practical caregiving strategies for families at home [11,30].

Community practitioners play an important role in reinforcing prophylactic iron supplementation (2–4 mg/kg/day) and underscoring that regular screening remains a key component of follow-up to prevent permanent neurodevelopmental sequelae [17,18]. Since gastrointestinal side effects of iron supplementation (such as constipation, dark stools, or feeding discomfort) may reduce parental adherence, anticipatory guidance from community professionals can help families manage these symptoms and support continued supplementation [14,17]. By doing so, they empower parents to safely continue supplementation rather than abandoning it out of fear or frustration [3,14].

Effective growth assessment extends beyond simple weight tracking and includes the use of specialised growth charts corrected for gestational age, together with careful parental guidance to balance the higher caloric intake required for catch-up growth against the long-term risks of rapid infant weight gain, which has been associated with an increased risk of future metabolic syndrome and cardiovascular disease [17,18,34]. In this context, community providers should encourage the continuation of expressed breastmilk or direct breastfeeding where possible, as its unique metabolic profile is highly protective against later adiposity compared with formula feeding [17,34]. By supporting evidence-based nutritional choices and appropriate growth monitoring, community professionals leverage the infant’s developmental “plasticity” [4] to support optimal long-term cardiometabolic health, proving that community follow-up is far more than merely treating short-term weight deficits.

### 3.5. Follow-Up of Infants with Complex Neonatal Conditions

Although the principles of coordinated community follow-up apply to all preterm infants, those discharged with complex neonatal conditions require substantially greater surveillance intensity and individualised care planning. Many preterm infants are discharged with ongoing respiratory, neurological, gastrointestinal, or technological care needs that require coordinated, individualised surveillance extending beyond routine post-discharge care [4,22,38]. Community health nurses, midwives, and health visitors are well positioned to provide continuity of care by translating specialist recommendations into practical home-based care, identifying early signs of deterioration, and facilitating timely referral when concerns arise [3,11,15].

Infants with bronchopulmonary dysplasia (BPD) represent one of the largest groups requiring enhanced community surveillance after discharge. Persistent respiratory vulnerability, prolonged oxygen dependency, and increased susceptibility to viral respiratory infections contribute substantially to rehospitalisation during infancy [7,12,39]. Consequently, community healthcare professionals should move beyond routine observation to undertake structured respiratory assessments during home visits, reinforce adherence to prescribed oxygen therapy where applicable, promote smoke-free home environments, support timely immunisation, and educate caregivers to recognise early indicators of respiratory deterioration, including increased work of breathing, feeding difficulties associated with respiratory distress, cyanosis, or reduced oxygen saturation when home monitoring is prescribed [14,39]. Close communication with neonatal and paediatric respiratory services is essential to support safe oxygen weaning and timely reassessment when respiratory status changes.

Infants discharged following necrotising enterocolitis (NEC), gastrointestinal surgery, or with enteral feeding devices present additional challenges that require close nutritional and clinical surveillance [17,34,40]. Community professionals play a pivotal role in monitoring feeding tolerance, hydration status, stoma or gastrostomy care where appropriate, and growth using corrected-age growth charts, while reinforcing prescribed nutritional supplementation and recognising early signs of feeding intolerance, poor weight gain, dehydration, or gastrointestinal complications requiring specialist review [17,41,42]. Home visits also provide opportunities to strengthen parental confidence in feeding practices, address practical concerns, and reinforce individualised nutritional plans established before discharge.

Similarly, infants with significant neurological vulnerability, including severe intraventricular haemorrhage, hypoxic-ischaemic encephalopathy, neonatal seizures, or evolving motor impairment, require ongoing developmental surveillance throughout infancy [4,32,43]. Although definitive neurological assessment remains the responsibility of specialist services, community professionals are often the first to observe subtle changes during repeated home assessments, including abnormalities in muscle tone, spontaneous movement, feeding coordination, visual attention, behavioural regulation, or caregiver-infant interaction [11,15,32]. Early recognition of these concerns facilitates timely referral to developmental paediatricians, physiotherapists, occupational therapists, and early intervention programmes, where prompt multidisciplinary management has been associated with improved developmental outcomes [4,32,43].

An increasing proportion of NICU graduates are also discharged with medical technologies, including home oxygen, nasogastric or gastrostomy feeding, cardiorespiratory monitors, and complex medication regimens [20,38,44]. Successful transition to home depends not only on technical competence but also on sustained family-centred support and coordinated multidisciplinary care. Community nurses, midwives, and health visitors can reinforce caregiver confidence through repeated demonstration of equipment use, medication review, troubleshooting of common technical problems, and development of emergency action plans tailored to the home environment [3,11,15,44]. Regular home visits additionally provide opportunities to assess caregiver burden, identify barriers to treatment adherence, and coordinate communication between primary care providers, neonatal specialists, therapists, and social services, thereby reducing fragmentation of care and supporting continuity across healthcare settings [11,28,44].

Across these diverse clinical conditions, the common determinant of successful community follow-up is not the underlying diagnosis itself but the ability to deliver proactive, coordinated, family-centred care that anticipates complications before hospital readmission becomes necessary [45]. Rather than adopting a uniform follow-up pathway for all preterm infants, community services should employ risk-stratified models of follow-up, recognising that infants with complex neonatal conditions require greater surveillance intensity, closer multidisciplinary collaboration, and more frequent reassessment than those with uncomplicated neonatal courses [4,14,38]. Accordingly, risk-stratified community follow-up should combine vigilant clinical surveillance, caregiver education, care coordination, and timely referral [14,31,32]. Within this framework, community nurses, midwives, and health visitors play a central role in supporting continuity of care and improving outcomes for infants with complex neonatal conditions.

## 4. Discussion

The journey of the preterm infant extends far beyond the NICU door. Although survival rates have improved substantially, the post-discharge period remains a vulnerable trajectory fraught with medical complexity and psychosocial stress [7,10,11]. Within this context, community-based, family-centred follow-up emerges not only as a clinical necessity but also as an ethical and social imperative [4,5]. As the present review highlights, improvements in survival should be matched by sustained support to optimise long-term outcomes [4].

Across the diverse literature reviewed, a unifying message emerges: effective post-discharge follow-up is inherently relational, proactive, and preventive, rather than merely clinical and episodic [3]. Collectively, the reviewed evidence suggests that the contribution of community nursing and midwifery within multidisciplinary follow-up can be conceptualised across four complementary practice domains: (1) clinical surveillance, (2) care coordination, (3) family-centred support, and (4) continuity of care. Although the scope of practice varies internationally, community nurses, midwives and health visitors consistently contribute across these domains by translating specialist neonatal care into coordinated, family-centred care within the home environment and facilitating timely referral when additional expertise is required. More specifically, community midwives often contribute most prominently during the immediate post-discharge transition, supporting neonatal adaptation alongside maternal recovery [3,11,35], whereas community and specialised paediatric nurses more commonly provide longitudinal developmental surveillance and ongoing management of infants with complex healthcare needs [3,5,11,15]. Together, these complementary roles strengthen continuity of care and reinforce multidisciplinary follow-up throughout infancy.

Successful models, whether employing structured transition tools such as Train-to-Home [2,14] or sustained home visiting [21,29], share distinct characteristics and consistently incorporate the four complementary practice domains identified in this review. They transcend basic medical surveillance by coaching parents, fostering caregiver autonomy, and establishing seamless, bidirectional communication between tertiary and primary care [11,30]. Ultimately, successful interventions do not simply monitor the infant; they actively build parental capacity and preparedness [8,46]. Increasingly, the evidence also suggests that follow-up should be tailored to the infant’s level of medical complexity, with risk-stratified models enabling more intensive surveillance and multidisciplinary support for infants with ongoing respiratory, neurological, nutritional, or technological care needs [4,14,38].

However, critical appraisal of the current evidence base reveals significant methodological limitations and knowledge gaps. Much of the existing literature relies on highly heterogeneous interventions, small sample sizes, and remarkably high attrition rates, which severely limit the generalisability of findings [3]. Furthermore, while the short-term benefits of home visiting on parental stress and confidence are well documented, data on the long-term impact on objective neurodevelopmental and clinical outcomes remain conflicting. It remains unclear which specific intervention components, or combination of the complementary practice domains identified in this review, constitute the true “active ingredients” of successful models. Similarly, the optimal dosage, visit frequency, and the professional composition of community follow-up teams remain insufficiently defined [3,21]. There is also a distinct lack of robust economic evaluations to demonstrate cost-effectiveness [3], alongside a pressing need to understand how these models can be equitably scaled to overcome profound disparities in access for rural or socioeconomically disadvantaged families [4,13,23].

To achieve these outcomes and mitigate avoidable healthcare use, healthcare systems should rethink service delivery to better support integrated, community-based follow-up. The persistent heterogeneity in resource allocation and the frequent disconnect between the NICU and community practice are not merely administrative flaws; they are important barriers to patient safety and continuity of care [3,4,31]. The available evidence supports a shift from siloed operations towards integrated care frameworks. This requires the adoption of standardised reception protocols, shared electronic health records, and formalised referral and care pathways [4,14]. Equally important is sustained investment in the education and professional development of community nurses, midwives, and health visitors, ensuring they have the competencies, resources, and organisational support required to deliver coordinated, evidence-based follow-up for preterm infants [3,11,15]. Integrated care pathways should also incorporate risk-stratified models of follow-up, allowing surveillance intensity to reflect the infant’s ongoing medical complexity rather than applying uniform follow-up schedules [4,14,38].

The increasing survival of medically complex NICU graduates further reinforces the need to move beyond uniform models of community follow-up. Infants discharged with chronic respiratory disease, neurological impairment, enteral feeding requirements, or home medical technologies require closer surveillance, coordinated multidisciplinary management, and greater support for caregivers than infants with uncomplicated neonatal courses [4,22,38,44]. Accordingly, future community follow-up services should adopt risk-stratified models that align surveillance intensity and multidisciplinary input with each infant’s clinical complexity while maintaining family-centred care and continuity across healthcare settings [38,45,47].

Within these risk-stratified models of community follow-up, endocrine and metabolic surveillance deserves greater attention than it currently receives. Despite their important long-term implications, endocrine and metabolic vulnerabilities remain comparatively under-recognised within routine community follow-up pathways [4]. Alongside neurodevelopmental monitoring, sustained clinical vigilance for conditions such as metabolic bone disease and anaemia of prematurity remains essential to prevent permanent sequelae [17,18,34]. The available evidence supports integrating routine endocrine and metabolic surveillance into standardised community follow-up pathways, highlighting the importance of developmentally informed, longitudinal care [4,17].

This review’s findings have several practical implications for community nursing and midwifery practice. First, community follow-up services should adopt integrated, risk-stratified models of care that reflect the evolving needs of preterm infants rather than relying on uniform follow-up pathways. Second, community nurses, midwives and health visitors require ongoing professional development to strengthen competencies in clinical surveillance, family-centred support, care coordination, and the early recognition of endocrine, metabolic, developmental, and other complications associated with prematurity [3,11,15,17]. Finally, healthcare systems should strengthen collaboration between neonatal services, primary care, and the wider multidisciplinary team through clear referral pathways, shared care plans, and effective communication systems, enabling community nurses, midwives, and health visitors to deliver coordinated, evidence-based follow-up while supporting families throughout the transition from hospital to home [3,4,14,32].

## 5. Conclusions

Community-based follow-up represents a critical extension of neonatal intensive care and is fundamental to improving the long-term health and well-being of preterm infants and their families. This narrative review highlights that effective post-discharge care extends beyond routine monitoring and is underpinned by four complementary practice domains: clinical surveillance, care coordination, family-centred support, and continuity of care. Within this framework, endocrine and metabolic surveillance and risk-stratified follow-up emerge as essential components of comprehensive community care tailored to each infant’s ongoing medical complexity. Within multidisciplinary follow-up models, community health nurses, midwives, and health visitors play a pivotal role in translating specialist recommendations into practical home-based care, strengthening caregiver confidence, and facilitating early recognition of complications.

Optimising outcomes requires integrated, evidence-informed follow-up, supported by stronger collaboration between neonatal and community services, structured communication, coordinated transfer-of-care pathways, and sustained investment in workforce development. By reducing service fragmentation and strengthening continuity across healthcare settings, these approaches have the potential to improve patient safety, support healthier childhood trajectories, and optimise long-term outcomes for preterm infants and their families.

From an educational perspective, preparing community nurses and midwives to manage the complex and evolving needs of preterm infants requires ongoing professional development, including training in clinical surveillance, developmental follow-up, care coordination and family-centred support.

Future research should identify which combinations of these complementary practice domains constitute the key components of effective community follow-up models, evaluate their clinical outcomes and cost-effectiveness, and explore strategies to improve equitable access to high-quality post-discharge services for preterm infants and their families.

## Data Availability

No new data were created or analyzed in this study.

## Abbreviations

The following abbreviations are used in this manuscript:

**BPD**	Bronchopulmonary dysplasia
**LCHD**	Life Course Health Development
**NEC**	Necrotising enterocolitis
**NICU**	Neonatal Intensive Care Unit
**SPIBI**	Stockholm Preterm Interaction-Based Intervention
**RSV**	Respiratory Syncytial Virus
**TSH**	Thyroid-Stimulating Hormone
